# Safety and efficacy of retreatment with immune checkpoint inhibitors after severe immune-related adverse events

**DOI:** 10.1093/oncolo/oyaf120

**Published:** 2025-06-14

**Authors:** Kazuyuki Mizuno, Takanori Ito, Tsunaki Sawada, Tomoko Kobayashi, Shintaro Iwama, Shoichiro Mori, Tetsunari Hase, Yuki Fukami, Kenji Furusawa, Yoshimitsu Yura, Ryota Morimoto, Ai Fujita Sajiki, Hiroaki Ushida, Noritoshi Kato, Shoichi Maruyama, Toyoaki Murohara, Masahisa Katsuno, Makoto Ishii, Masashi Akiyama, Hiroshi Arima, Hiroki Kawashima, Yuichi Ando

**Affiliations:** Department of Clinical Oncology and Chemotherapy, Nagoya University Hospital, Nagoya, Japan; Department of Gastroenterology and Hepatology, Nagoya University Graduate School of Medicine, Nagoya, Japan; Department of Gastroenterology and Hepatology, Nagoya University Graduate School of Medicine, Nagoya, Japan; Department of Gastroenterology and Hepatology, Nagoya University Graduate School of Medicine, Nagoya, Japan; Department of Endocrinology and Diabetes, Nagoya University Graduate School of Medicine, Nagoya, Japan; Department of Endocrinology and Diabetes, Nagoya University Graduate School of Medicine, Nagoya, Japan; Department of Dermatology, Nagoya University Graduate School of Medicine, Nagoya, Japan; Department of Respiratory Medicine, Nagoya University Graduate School of Medicine, Nagoya, Japan; Department of Neurology, Nagoya University Graduate School of Medicine, Nagoya, Japan; Department of Cardiology, Nagoya University Graduate School of Medicine, Nagoya, Japan; Department of Cardiology, Nagoya University Graduate School of Medicine, Nagoya, Japan; Department of Cardiology, Nagoya University Graduate School of Medicine, Nagoya, Japan; Department of Ophthalmology, Nagoya University Graduate School of Medicine, Nagoya, Japan; Department of Ophthalmology, Nagoya University Graduate School of Medicine, Nagoya, Japan; Department of Nephrology, Nagoya University Graduate School of Medicine, Nagoya, Japan; Department of Nephrology, Nagoya University Graduate School of Medicine, Nagoya, Japan; Department of Cardiology, Nagoya University Graduate School of Medicine, Nagoya, Japan; Department of Neurology, Nagoya University Graduate School of Medicine, Nagoya, Japan; Department of Clinical Research Education, Nagoya University Graduate School of Medicine, Nagoya, Japan; Department of Respiratory Medicine, Nagoya University Graduate School of Medicine, Nagoya, Japan; Department of Dermatology, Nagoya University Graduate School of Medicine, Nagoya, Japan; Department of Endocrinology and Diabetes, Nagoya University Graduate School of Medicine, Nagoya, Japan; Department of Gastroenterology and Hepatology, Nagoya University Graduate School of Medicine, Nagoya, Japan; Department of Clinical Oncology and Chemotherapy, Nagoya University Hospital, Nagoya, Japan

**Keywords:** immune checkpoint inhibitors, immune-related adverse events, retreatment, safety, efficacy

## Abstract

**Background:**

While immune checkpoint inhibitors (ICIs) have revolutionized cancer treatment, they can trigger severe immune-related adverse events (irAEs). The safety and efficacy of ICI retreatment after severe irAEs remain poorly understood.

**Methods:**

We conducted a retrospective analysis of 1271 patients with malignancies treated with ICIs at a university hospital in Japan between September 2014 and June 2023. We evaluated the incidence and characteristics of severe irAEs, defined as grade ≥3, and the safety and efficacy of ICI retreatment.

**Results:**

Severe irAEs occurred in 222 patients (17.5%). Patients with single endocrinopathies were excluded, and 46 (28.4%) of the remaining 162 patients underwent ICI retreatment. Upon retreatment, 14 patients (30.4%) experienced recurrent or new grade ≥2 irAEs. One patient who experienced hepatotoxicity (grade 3) at initial ICI treatment developed a recurrence (grade 4). Regarding antitumor response, the objective response rate to retreatment was 28.3% (13/46), with 10.9% achieving complete and 17.4% partial response. The median duration of ICI administration after retreatment was 218 days (95% confidence interval [CI]: 84-399). At 1 year after retreatment, 15.4% (95% CI: 6.8-27.4) of patients discontinued due to irAEs, 44.4% (95% CI: 29.7-58.1) due to disease progression, 6.6% (95% CI: 1.7-16.3) completed planned treatment, and 33.4% (95% CI: 20.3-47.2) continued treatment.

**Conclusions:**

ICI retreatment after severe irAEs demonstrated a manageable safety profile and promising efficacy, even in patients with grade ≥3 irAEs. ICI retreatment may be a viable option for patients with limited alternatives, particularly those showing favorable antitumor responses at initial treatment.

## Graphical Abstract

**Graphical Abstract:**
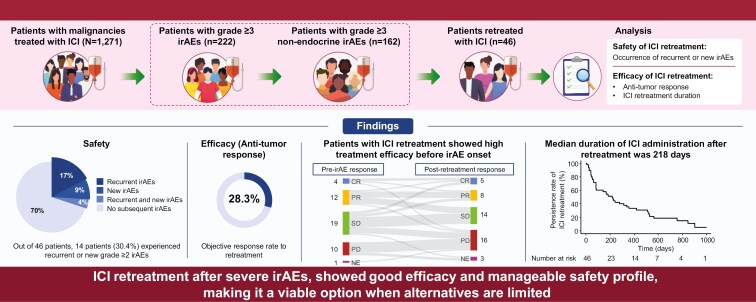

**Implications for Practice**

This study provides critical evidence-based guidance for physicians considering immune checkpoint inhibitor (ICI) retreatment after severe immune-related adverse events (irAEs). Our findings suggest that ICI retreatment can be safely implemented with a 30.4% risk of recurrent or new grade ≥2 irAEs while showing meaningful clinical responses (objective response rate: 28.3%). Careful patient selection through multidisciplinary consultation, particularly focusing on those who showed favorable initial responses and have limited alternative treatment options, is crucial for successful retreatment. Insights provided here will enable clinicians to make more informed decisions about ICI retreatment, potentially expanding treatment opportunities for patients with cancer who experienced severe irAEs.

## Introduction

Immune checkpoint inhibitors (ICIs) have revolutionized cancer treatment, demonstrating unprecedented efficacy against various advanced solid tumors and hematologic malignancies.^1-3^ These therapies, including anti-cytotoxic T-lymphocyte-associated protein 4 (CTLA-4),^4^ anti-programmed cell death protein 1 (PD-1),^5^ and anti-programmed death-ligand 1 (PD-L1) agents,^6^ have significantly improved patient outcomes through either monotherapy or combination approaches.

ICIs enhance antitumor immune responses by blocking inhibitory signaling pathways but can trigger immune-related adverse events (irAEs).^7^ These irAEs can affect multiple organ systems, commonly occurring in the endocrine glands, liver, gastrointestinal tract, and skin.^7^ Although less commonly, irAEs may also affect the central nervous system, cardiovascular system, lungs, and eyes. The management of irAEs is guided by their severity and the affected organ system.^8-11^ While endocrine irAEs often require hormone replacement therapy and may not necessitate ICI discontinuation, severe non-endocrine irAEs classified as grade 3 or higher generally warrant high-dose corticosteroids and temporary or permanent ICI discontinuation according to current guidelines.^8-11^

ICI retreatment after severe irAEs presents a complex clinical dilemma, balancing potential benefits against the risk of irAE recurrence.^12^ A previous study using the WHO VigiBase reported a 28.8% irAE recurrence rate in the same organ after ICI retreatment. However, this study lacked detailed severity grading, provided limited follow-up information, and had potential heterogeneity in patient populations.^13^ Furthermore, comprehensive data regarding the efficacy of ICI retreatment are scarce.

To address this critical knowledge gap, several studies, including meta-analyses, have investigated the safety and efficacy of ICI retreatment. One systematic review and meta-analysis of 789 ICI retreatment cases found a pooled all-grade and high-grade (≥grade 3) irAE recurrence rate of 34.2% and 11.7%, respectively.^14^ Another review of 31 studies involving 812 patients suggested that ICI retreatment could be beneficial, especially when the initial ICI discontinuation was not due to disease progression.^15^ However, these meta-analyses highlight limitations in existing data, including study heterogeneity and incomplete reporting of patient characteristics, treatment regimens, and irAE management. Therefore, we conducted a retrospective analysis focusing on severe irAEs and outcomes after ICI retreatment to provide further insight and inform clinical decision-making in patients with advanced malignancies.

## Methods

### Patient selection

This retrospective study included patients treated with ICIs for malignancies at Nagoya University Hospital between September 2014 and June 2023. Eligible patients received at least one cycle of PD-1/PD-L1 inhibitors and/or CTLA-4 inhibitors, either as monotherapy or in combination with other drugs.

### Data collection

Clinical data collected from medical records included demographics, cancer type, ICI details, treatment duration, treatment outcomes, and irAE characteristics (onset, affected organs, grade, and steroid use). Adverse events were graded using the common terminology criteria for adverse events version 5.0, with a grade ≥3 defined as severe.

Although irAEs were originally recorded as part of routine clinical care, all potential cases were retrospectively reviewed by a multidisciplinary team of organ-specific specialists. This review primarily focused on cases of suspected irAEs or those with missing grade information. The review aimed to confirm the diagnosis and ensure consistent grading according to common terminology criteria for adverse events v5.0, referencing the European Society for Medical Oncology (ESMO), American Society of Clinical Oncology (ASCO), and Japanese Society of Medical Oncology (JSMO) guidelines.^8,9,11^

PD-1 and PD-L1 inhibitors were grouped together in this analysis because they target the same immune checkpoint pathway and have demonstrated similar efficacy and toxicity profiles in previous studies.^13,14^

Time to irAE onset was defined as the number of days from the start of the causative ICI administration to grade ≥3 irAEs. Since ICI retreatment may be contraindicated in the case of grade 3 irAEs, the initial ICI course was targeted at patients with grade ≥3 irAEs. For retreatment cases, time to irAE onset was measured from the start of ICI retreatment to grade ≥2 irAEs, as these are generally considered clinically significant and may require intervention or treatment modification.

The final follow-up of medical records was conducted between February and May 2024, ensuring a minimum observation period of 7 months from initial treatment.

This study was conducted in accordance with the Declaration of Helsinki (2013 revision) and approved by the Ethics Committee of Nagoya University Hospital (IRB No. 2023-0207).

### Safety and efficacy of ICI retreatment

This retrospective study did not follow a prospective protocol for ICI or irAE management. Treatment decisions—including initial ICI discontinuation and subsequent retreatment—were based on the treating physician’s clinical judgment in accordance with established guidelines (eg, ASCO, ESMO, JSMO) and multidisciplinary consultations at the time of treatment. In clinical practice, ICI retreatment was offered to patients with severe irAEs when physicians judged that the potential benefits justified the risks and alternative treatment options were limited.

In this study, “retreatment” is defined as any administration of an ICI to a patient who had previously experienced a severe irAE, regardless of the type of ICI used for retreatment, and without any predefined drug holiday period after the resolution of the irAE.

According to guidelines, continuation or retreatment with ICIs is generally feasible for endocrine-related irAEs, provided that appropriate hormone replacement therapy is in place and the patient’s condition is stable. Therefore, we focused on grade ≥3 non-endocrine irAEs for the retreatment analysis.

Efficacy was evaluated based on the best overall response according to the Response Evaluation Criteria in Solid Tumors version 1.1. Safety was assessed by monitoring the incidence, severity, and manageability of recurrent or new irAEs during the retreatment period.

### Statistical analysis

Continuous variables were described as median [interquartile range (IQR)] and compared using t- or Mann–Whitney *U* tests. Categorical variables were compared using chi-square or Fisher’s exact tests. Kaplan–Meier and competing risks analyses were used for retreatment duration and discontinuation, respectively. For all statistical tests, a *P*-value of <.05 was considered statistically significant.

All statistical analyses were performed with EZR (Saitama Medical Center, Jichi Medical University, Saitama, Japan), a graphical user interface for R (The R Foundation for Statistical Computing, Vienna, Austria). EZR is a modified version of R Commander designed to add statistical functions frequently used in biostatistics.^16^

## Results

### Characteristics of patients with severe irAEs (grade ≥3)

**Figure 1.**
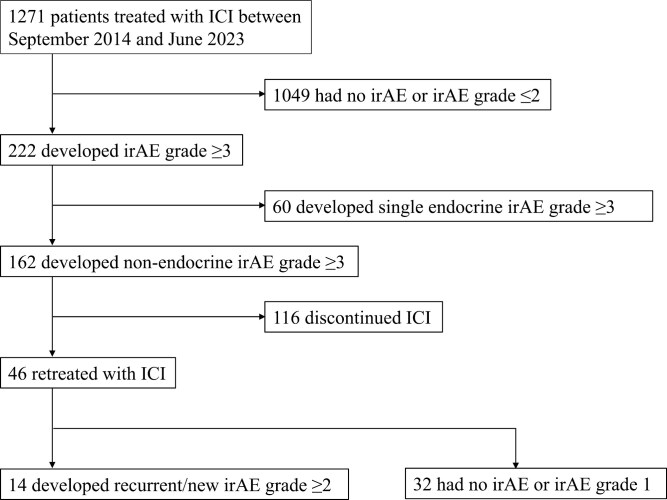
Flowchart of the patient selection process. ICI, Immune checkpoint inhibitor; irAE, Immune-related adverse event.

A total of 1271 patients received ICI treatment at a university hospital in Japan between September 2014 and June 2023. Among these, 222 patients (17.5%) experienced grade ≥3 irAEs (Figure 1). These irAEs included endocrinopathies (*n* = 75, 5.9%), hepatotoxicity (*n* = 71, 5.6%), dermatitis (*n* = 29, 2.3%), colitis (*n* = 26, 2.0%), pneumonitis (*n* = 23, 1.8%), pancreatitis (*n* = 10, 0.8%), neuropathy (*n* = 9, 0.7%), myocarditis (*n* = 6, 0.4%), uveitis (*n* = 4, 0.3%), and nephritis (*n* = 2, 0.2%). Among the 222 patients with grade ≥3 irAEs, 30 (13.5%) experienced events in multiple organ systems. The most severe irAE for each patient was grade 3 in 193 patients (15.2%), grade 4 in 27 patients (2.1%), and grade 5 in 2 patients (0.2%; Table 1). Both grade 5 events were due to pneumonitis.

**Table 1.** Characteristics of patients with severe and mild/no immune-related adverse events following immune checkpoint inhibitor therapy.

Characteristics	Severe irAEs	Mild/no irAEs	*P*-value
	(*N* = 222)	(*N* = 1049)	
**Age, year, median (IQR)**	67 (58–73)	69 (59–75)	.029
**Sex, *n* (%)**			.339
Female	75 (33.8)	320 (30.5)	
Male	147 (66.2)	729 (69.5)	
**Initial ICI type, *n* (%)**			<.001
Anti-PD-(L)1	176 (79.3)	1003 (95.6)	
Anti-CTLA-4	6 (2.7)	6 (0.6)	
Anti-PD-(L)1 and anti-CTLA-4 combination	40 (18.0)	40 (3.8)	
**First ICI regimen, *n* (%)**			.413
ICI only	163 (73.4)	746 (71.1)	
ICI + Chemotherapy	40 (18.0)	189 (18.0)	
ICI + TKI	11 (5.0)	84 (8.0)	
ICI + Chemotherapy + TKI	8 (3.6)	30 (2.9)	
**Tumor type, *n* (%)**			<.001
Lung cancer and malignant pleural mesothelioma	74 (33.3)	345 (32.9)	
Gastrointestinal cancer	24 (10.8)	179 (17.1)	
Malignant melanoma and other skin cancer	62 (27.9)	101 (9.6)	
Renal cancer and urothelial carcinoma	30 (13.5)	130 (12.4)	
Head and neck cancer	14 (6.3)	137 (13.1)	
Others	18 (8.1)	157 (15.0)	
**Type of irAE grade ≥3, *n* (%)**			
Endocrinopathies	75 (33.8)	N/A	
Hepatotoxicity	71 (32.0)	N/A	
Dermatitis	29 (13.1)	N/A	
Colitis	26 (11.7)	N/A	
Pneumonitis	23 (10.4)	N/A	
Pancreatitis	10 (4.5)	N/A	
Neuropathy	9 (4.1)	N/A	
Myocarditis	6 (2.7)	N/A	
Uveitis	4 (1.8)	N/A	
Nephritis	2 (0.9)	N/A	
**Highest irAE grade, *n* (%)**			
Grade 3	193 (86.9)	N/A	
Grade 4	27 (12.2)	N/A	
Grade 5	2 (0.9)	N/A	

Severe irAEs are defined as grade ≥3, while non-severe irAEs include grade ≤2 or no irAEs. N/A, not applicable (for the non-severe group).

Statistical tests: Chi-square test or Fisher’s exact test for categorical variables, and Mann–Whitney *U* test for continuous variables.

CTLA-4, cytotoxic T-lymphocyte-associated protein 4; Chemotherapy, cytotoxic chemotherapy; IQR, interquartile range; ICI, immune checkpoint inhibitor; irAE, immune-related adverse event; PD-1, programmed cell death protein 1; PD-L1, programmed cell death ligand 1; TKI, tyrosine kinase inhibitor.

Table 1 compares the clinical characteristics of patients who experienced grade ≥3 irAEs to those who did not. Patients with severe irAEs were significantly younger (median age 67 vs. 69 years, *P* = .029). Among patients with severe irAEs, 46 out of 222 (20.7%) received anti-CTLA-4 agent-containing regimens as initial ICI treatment, as compared to 46 out of 1049 (4.4%) in the mild/no irAE group (*P* < .001). Additionally, a higher proportion of patients with malignant melanoma and other skin cancers experienced severe irAEs (27.9% vs. 9.6%, *P* < .001).

**Figure 2.**
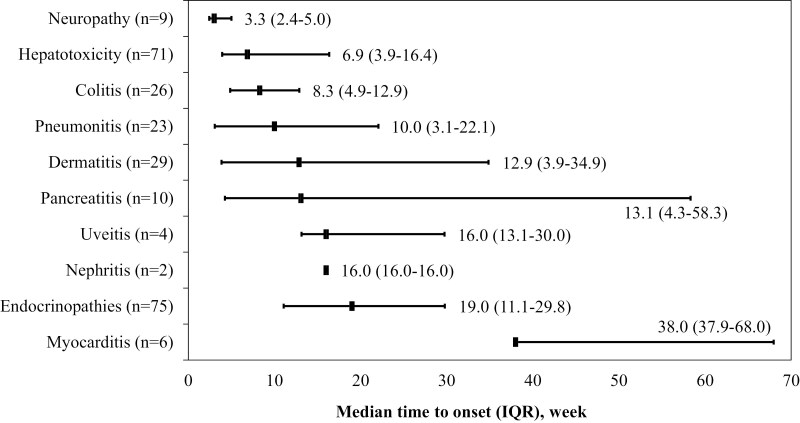
Median time to onset of grade 3/4 immune-related adverse events following immune checkpoint inhibitor therapy. IQR, Interquartile range.

Figure 2 illustrates the timing of onset for grade ≥3 irAEs in initial ICI treatment by the organ system. Neuropathy occurred earliest with a median onset of 3.3 (IQR 2.4-5.0) weeks, followed by hepatotoxicity at 6.9 (IQR 3.9-16.4) weeks and colitis at 8.3 (IQR 4.9-12.9) weeks. In contrast, endocrinopathies had a later onset with a median of 19.0 (IQR 11.1-29.8) weeks, while myocarditis showed the latest onset at 38.0 (IQR 11.3-64.1) weeks.

### Characteristics of patients retreated after severe irAEs

We analyzed 162 patients with severe irAEs, excluding those with grade ≥3 endocrine irAEs alone, which can be continued with ICI treatment with hormone replacement. However, we included 15 who had severe non-endocrine irAEs concurrent with endocrine irAEs (eg, multisystem irAE). Of these, 46 patients (28.4%) were retreated with ICIs (Figure 1).

Table 2 summarizes the characteristics of patients who underwent ICI retreatment after experiencing severe irAEs. Patients who received ICI retreatment were significantly more likely to have been initially treated with a combination of anti-PD-1 or anti-PD-L1 and anti-CTLA-4 therapies (*P* = .016). Among retreated patients, those with hepatotoxicity as their initial irAE were significantly more common (*P* = .022), while those with pneumonitis were significantly less (*P* = .005). Notably, none of the patients who developed myocarditis were retreated.

**Table 2.** Comparison of characteristics between patients who received immune checkpoint inhibitors retreatment and those who discontinued treatment after experiencing immune-related adverse events.

Characteristics	Retreat	Discontinue	*P*-value
	(*N* = 46)	(*N* = 116)	
**Age, year, median (IQR)**	64 (57–73)	66 (57–73)	.302
**Sex, *n* (%)**			.358
Female	18 (39.1)	36 (31.0)	
Male	28 (60.9)	80 (69.0)	
**ICI type that induced initial irAE, *n* (%)**			.016
Anti-PD-(L)1	26 (56.5)	91 (78.4)	
Anti-CTLA-4	4 (8.7)	4 (3.4)	
Anti-PD-(L)1 and anti-CTLA-4 combo	16 (34.8)	21 (18.1)	
**Tumor type, *n* (%)**			.098
Lung cancer and malignant pleural mesothelioma	10 (21.7)	42 (36.2)	
Malignant melanoma and other skin cancer	19 (41.3)	27 (23.3)	
Renal cancer and urothelial carcinoma	10 (21.7)	16 (13.8)	
Gastrointestinal cancer	4 (8.7)	13 (11.2)	
Head and neck cancer	1 (2.2)	9 (7.8)	
Others	2 (4.3)	9 (7.8)	
**Type of irAE grade ≥**3, *n* (%)^*^			
Hepatotoxicity	27 (58.7)	44 (37.9)	.022
Dermatitis	6 (13.0)	21 (18.1)	.493
Colitis	6 (13.0)	19 (16.4)	.810
Pneumonitis	1 (2.2)	22 (19.0)	.005
Endocrinopathies^†^	6 (13.0)	9 (7.8)	.367
Pancreatitis	5 (10.9)	5 (4.3)	.149
Neuropathy	2 (4.3)	7 (6.0)	1.000
Myocarditis	0 (0.0)	6 (5.2)	.185
Uveitis	2 (4.3)	2 (1.7)	.319
Nephritis	2 (4.3)	0 (0.0)	.079
**Highest irAE grade, *n* (%)** ^‡^			.887
Grade 3	42 (91.3)	101 (87.1)	
Grade 4	4 (8.7)	13 (11.2)	
Grade 5	0 (0.0)	2 (1.7)	
**Steroid use for irAE management, *n* (%)**	23 (50.0)	96 (82.8)	<.001
**Second-line immunosuppressants, *n* (%)**	0 (0.0)	14 (12.1)	.004
**Time to irAE onset, day, median (IQR)**	55 (27-107)	54 (26-162)	.556
**Best response before irAE onset, *n* (%)**			
CR	4 (8.7)	4 (3.4)	.225
PR	12 (26.1)	24 (20.7)	.530
SD	19 (41.3)	40 (34.5)	.470
PD	10 (21.7)	33 (28.4)	.435
NE	1 (2.2)	4 (3.4)	1
Adjuvant	0 (0.0)	11 (9.5)	.035

^*^Patients may have experienced multiple types of grade ≥3 irAEs; therefore, the sum of percentages in this category may exceed 100%.

^†^Patients with grade ≥3 endocrinopathies were included in the analysis only if they had concurrent severe non-endocrine irAEs.

^‡^Highest irAE grade recorded for non-endocrine irAEs.

Statistical tests: Chi-square test or Fisher’s exact test for categorical variables, and Mann–Whitney U test for continuous variables.

CTLA-4, cytotoxic T-lymphocyte-associated protein 4; CR, complete response; IQR, interquartile range; IrAE, immune-related adverse event; ICI, immune checkpoint inhibitor; NE, not evaluable; PD-1, programmed cell death protein 1; PD-L1, programmed cell death ligand 1; PR, partial response; PD, progressive disease; SD, stable disease.

Retreated patients were significantly less likely to have required steroid treatment for their initial irAE (*P* < .001). In contrast, no patients received ICIs as adjuvant therapy after surgery. Time to onset of the initial irAE and the best antitumor response prior to irAE onset were not significant factors in determining whether retreatment was undertaken.

The median time from the last ICI administration before irAE onset to ICI retreatment was 70 days (IQR 42-139).

### Safety of retreatment with ICI after severe irAEs

**Figure 3.**
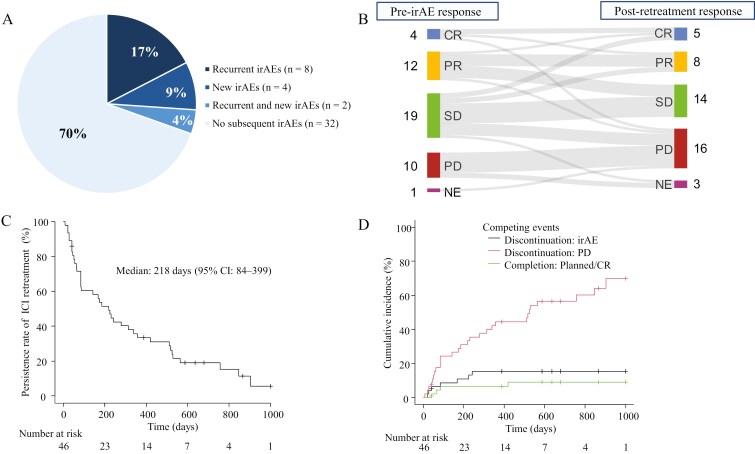
Outcomes of immune checkpoint inhibitor retreatment after initial immune-related adverse events. (A) Frequency of recurrent or new irAEs after ICI retreatment. (B) Comparison of best antitumor response before initial irAE and after ICI retreatment. (C) Duration of ICI administration after initiation of retreatment. (D) Competing risk analysis of causes for ICI discontinuation after retreatment. Abbreviations: CR, Complete response; CI, Confidence interval; ICI, Immune checkpoint inhibitor; irAEs, Immune-related adverse events; PR, Partial response; PD, Progressive disease; SD, Stable disease.

Among the 46 patients with severe non-endocrine irAEs who were retreated, 14 (30.4%) experienced recurrent or new grade ≥2 irAEs upon retreatment. Specifically, 8 patients (17.4%) experienced recurrent irAEs in the same organ, 2 (4.3%) had both recurrent and new irAEs, and 4 (8.7%) developed new irAEs (Figures 1 and 3A). Table 3 shows the comparison between patients who experienced irAE recurrence or new onset and those who did not develop irAEs after ICI retreatment. We found no significant differences in the initial irAE by ICI type, retreatment agent selection, grade of initial irAE, steroid use, or occurrence of multisystem irAEs. However, these results should be interpreted cautiously due to the limited sample size.

**Table 3.** Characteristics of patients with and without subsequent immune-related adverse events after immune checkpoint inhibitor retreatment.

Characteristics	Subsequent irAEs	No subsequent irAEs	*P*-value
	(*N* = 14)	(*N* = 32)	
**ICI type that induced initial irAE, *n* (%)**			.886
Anti-PD-(L)1	9 (64.3)	17 (53.1)	
Anti-CTLA-4	1 (7.3)	3 (9.4)	
Anti-PD-(L)1 and anti-CTLA-4 combination	4 (28.6)	12 (37.5)	
**Retreatment ICI, *n* (%)**			.893
Same ICI	10 (71.4)	20 (62.5)	
Different ICI	3 (21.4)	7 (21.9)	
Combo to PD-(L)1 mono	1 (7.1)	5 (15.6)	
**Highest grade of initial irAE, *n* (%)**			.887
Grade 3	12 (85.7)	30 (93.8)	
Grade 4	2 (14.3)	2 (6.2)	
**Steroid use for irAE management, *n* (%)**	7 (50.0)	16 (50.0)	1.000
**Initial multiple irAEs grade ≥3, *n* (%)**	4 (28.3)	5 (15.6)	.423

Statistical tests: Chi-square test or Fisher’s exact test for categorical variables.

CTLA-4, cytotoxic T-lymphocyte-associated protein 4; irAE, immune-related adverse event; ICI, immune checkpoint inhibitor; PD-1, programmed cell death protein 1; PD-L1, programmed cell death ligand 1.

Table 4 summarizes the cases of recurrent irAEs. Only one patient experienced a recurrence of hepatotoxicity upon retreatment, with the grade worsening from 3 initially to 4. This case was successfully managed with corticosteroid treatment and did not require second-line immunosuppressants. No deaths were attributed to irAEs induced by ICI retreatment.

**Table 4.** Outcomes of patients undergoing immune checkpoint inhibitor retreatment.

Age	Tumor type	Initial causative ICI	ICI retreatment	Time to initial irAE onset (days)	Initial irAE	Subsequent irAE	Retreatment duration (days)	Retreat discontinuation reason
50s	Malignant melanoma	Ipilimumab + Nivolumab	Pembrolizumab	42	Hepatotoxicity G4, Neuropathy G3	Neuropathy G2, Hypophysitis G3	42	Due to irAE
40s	Lung cancer	Atezolizumab	Atezolizumab	2	Hepatotoxicity G3	Hepatotoxicity G4	84	Due to irAE
60s	Lung cancer	Pembrolizumab	Pembrolizumab	415	Hepatotoxicity G3, Dermatitis G3	Dermatitis G3	224	Due to irAE
60s	Malignant melanoma	Ipilimumab + Nivolumab	Nivolumab	73	Hepatotoxicity G3, Colitis G3	Colitis G3	56	PD
40s	Malignant melanoma	Ipilimumab	Ipilimumab	34	Hepatotoxicity G3, Colitis G3	Hypophysitis G3	529	PD
80s	Renal cancer	Ipilimumab + Nivolumab	Ipilimumab + Nivolumab	35	Hepatotoxicity G3	Adrenal insufficiency G3, Hypothyroidism G2	357	PD
50s	Malignant melanoma	Ipilimumab + Nivolumab	Pembrolizumab	89	Hepatotoxicity G3	Pneumonitis G2	85	PD
70s	Renal cancer	Nivolumab	Nivolumab	42	Hepatotoxicity G3	Hepatotoxicity G2	185	PD
50s	Malignant melanoma	Nivolumab	Ipilimumab	90	Dermatitis G4	Dermatitis G2	86	Planned completion
80s	Lung cancer	Nivolumab	Nivolumab	152	Dermatitis G3	Dermatitis G2	142	PD
70s	Esophageal cancer	Nivolumab	Nivolumab	963	Dermatitis G3	Pneumonitis G2, Dermatitis G2	168	Due to irAE
60s	Lung cancer	Nivolumab	Nivolumab	16	Pneumonitis G3	Pneumonitis G3	28	Due to irAE
60s	Urothelial carcinoma	Pembrolizumab	Pembrolizumab	193	Colitis G3	Dermatitis G3	242	Due to irAE
70s	Head and neck cancer	Nivolumab	Nivolumab	112	Nephritis G3	Hypophysitis G3	867	Ongoing

ICI, immune checkpoint inhibitor; irAE, immune-related adverse event; G2, G3, G4, grade 2, 3, 4 (severity of adverse events); PD, progressive disease.

### Efficacy of retreatment with ICI after initial irAEs

After ICI retreatment, 5 (10.9%) achieved complete response (CR), 8 (17.4%) had partial response (PR), 14 (30.4%) showed stable disease (SD), and 16 (34.8%) had progressive disease (PD; Figure 3B). The profiles of 5 patients who achieved CR are provided in the Supplementary Table.

Next, we compared the best treatment response before initial irAE onset with the antitumor effect during retreatment (Figure 3B). Among patients evaluable for response after ICI retreatment, those with the best overall response of CR or PR prior to the initial irAE were significantly more likely to achieve a CR or PR following retreatment than those with SD or PD (9/16 [56.3%] vs. 4/26 [15.4%], *P* = .014). Furthermore, a significantly higher proportion of patients with a pre-irAE best overall response of CR, PR, or SD maintained disease control (27/34 [79.4%]) than those with a pre-irAE best response of PD (0/8; *P* < .001).

The median duration of ICI treatment after retreatment was 218 days (95% confidence interval [CI]: 84-399; Figure 3C). The median overall survival was 665 days (95% CI: 443-929), and the median progression-free survival was 178 days (95% CI: 70-301).

Using a competing risk model, we analyzed the reasons for treatment discontinuation at one year: 15.4% (95% CI: 6.8-27.4) of patients discontinued due to irAEs, 44.4% (95% CI: 29.7-58.1) due to PD or deterioration of general condition, 6.6% (95% CI: 1.7-16.3) due to planned completion or CR, and 33.4% (95% CI: 20.3-47.2) were still continuing treatment (Figure 3D).

## Discussion

This comprehensive retrospective study of 1271 ICI-treated patients provides crucial insights into the safety and efficacy of ICI retreatment following severe irAEs. Among 162 cases with grade ≥3 non-endocrine irAEs, 46 patients (28.4%) underwent ICI retreatment. Our findings revealed that while approximately 30% experienced recurrent or new irAEs, most were clinically manageable, and importantly, we observed meaningful objective responses to the retreatment.

The evaluation of ICI retreatment safety has been historically limited by the exclusion of patients with prior severe irAEs from clinical trials,^12^ hindering the understanding of real-world outcomes. A WHO VigiBase analysis included 452 ICI retreatments and a 28.8% irAE recurrence rate was reported (95% CI: 24.8-33.1),^13^ but the study lacked detailed severity data and focused on same-agent retreatments.

Meta-analyses have also found varying irAE recurrence rates with ICI retreatment, suggesting potential benefit, particularly when initial ICI discontinuation was not due to disease progression.^14,15^ However, these analyses were limited by heterogeneity and incomplete reporting. Our study addresses this gap by examining retreatment outcomes in patients with severe (grade ≥3) irAEs during initial treatment, a population often excluded from trials. This challenging scenario requires carefully balancing therapeutic benefits and safety risks. By analyzing cases in which patients with limited options underwent retreatment after thorough counseling, our findings offer real-world evidence for clinical decision-making.

Liver irAEs were the most common type in our ICI retreatment cohort, accounting for 58.7% (27/46) of all retreatment cases and 38.0% (27/71) of all grade 3 or 4 hepatotoxicity cases. For grade 3-4 hepatotoxicity, ASCO guidelines recommend permanent discontinuation.^8^ ESMO guidelines recommend permanent discontinuation but allow anti-PD-1 or anti-PD-L1 monotherapy retreatment for those previously on combination therapy.^9^ NCCN Guidelines 2024 recommend permanent discontinuation for grade 4 synthetic dysfunction or biliary strictures, but temporary hold for grade 3 cases.^10^

Our analysis showed that a small proportion of patients experienced recurrent hepatotoxicity or other irAEs upon retreatment, with hepatotoxicity manageable with corticosteroids. Hountondji et al. reported that 12 out of 51 patients (23.5%) experienced grade ≥2 hepatotoxicity recurrence during ICI retreatment.^17^ These results differ from the frequency of hepatotoxicity relapse observed in our study, which might be related to sample size or the fact that patients with severe, life-threatening irAEs, such as immune-related sclerosing cholangitis, were generally not considered for ICI retreatment in our clinical practice.

In contrast, retreatment rates for irAE pneumonitis and myocarditis were low at 4.3% (1/23) and 0.0% (0/6), respectively. These irAEs can be potentially life-threatening, with a high risk of recurrence upon retreatment, and there is limited data available on ICI retreatment for these conditions.^18-20^ These retreatment rates are consistent with guidelines, which unanimously recommend discontinuation for grade ≥3 pneumonitis.^8-10^ Similarly, for myocarditis, ASCO and NCCN recommend discontinuation for grades≥2,^8,10^ while ESMO generally recommends permanent discontinuation.^9^ The high retreatment risk likely explains the low retreatment rates observed in clinical practice.

Notably, retreated patients were significantly less likely to have required steroid treatment for their initial irAE. This might appear counterintuitive, as both groups exhibited similar rates of grade ≥3 events. However, this difference likely reflects a selection bias in real-world clinical practice: patients whose irAEs were successfully managed without high-dose steroids (eg, some cases of pancreatitis or grade 3 hepatotoxicity that improved without steroid intervention^21,22^) might have been perceived as lower-risk candidates for retreatment. This highlights the importance of a careful, individualized decision-making process when considering ICI retreatment, weighing potential benefits against risks.

The safety profile of ICI retreatment after severe non-endocrine irAEs in our study was generally favorable, as 69.6% of patients avoided grade ≥2 recurrent or new irAEs. Grade ≥3 irAE incidence after retreatment (9/46, 19.6%) was comparable to the initial irAE occurrence (222/1,271; 17.5%; (*P* = .694). While these results indicate that safe retreatment is possible with careful patient selection, the 30% rate of grade ≥2 irAEs emphasizes a requirement for close monitoring during retreatment.

Regarding antitumor efficacy, our study provides valuable information on the potential benefit of ICI retreatment. The observed response and disease control rates suggest that ICI retreatment should not be dismissed as a treatment option. We found that a favorable initial response to ICI was a significant predictor of response to retreatment. Meanwhile, patients who experienced PD as their best response during initial treatment were likely to have PD upon retreatment, suggesting limited efficacy in this subgroup. In addition, the relatively long median duration of ICI administration after retreatment, coupled with a low discontinuation rate due to irAEs, suggests that long-term treatment continuation was achievable. These results indicate that patients with a good initial antitumor response may benefit from ICI retreatment when treatment options are limited.

Myocarditis showed the latest onset among irAEs with a median of 38.0 weeks, which is longer than typically reported in the literature. For example, in their recent study, Gougis et al. reported a median onset time of 31 days (IQR 21-67) for myositis.^23^ Possible explanations for this discrepancy include the small number of myocarditis cases in our cohort, differences in patient populations, and variations in monitoring practices. Further research with larger cohorts is needed to confirm this finding and better elucidate the factors influencing the timing of myocarditis onset after ICI treatment.

Our study’s strength is its consistent treatment policy and thorough medical record review at a single institution. However, our study has some limitations. First, it may lack statistical power due to its retrospective nature and small sample size. Second, the retrospective nature of this study might have led to the underreporting of mild irAEs, as these are not always documented in medical records. Third, we were unable to assess the safety of retreatment for potentially fatal irAEs such as cardiac, neurological, and pulmonary toxicities due to the limited number of cases. Finally, while the median time from the last ICI dose to retreatment was 70 days, irAEs can persist beyond 90 days post-ICI.^24^ This means that some irAEs observed after retreatment might have been continuations of the initial event, complicating causality. Retreatment could also exacerbate pre-existing, subclinical irAEs. Therefore, our findings regarding retreatment safety and efficacy should be interpreted cautiously, and prospective studies are needed to define the optimal timing for ICI retreatment.

Clinicians should consider these safety and efficacy data in ICI retreatment decisions. Large prospective trials are needed for low-risk irAEs like hepatotoxicity, while retrospective data analysis should continue for high-risk cases to better assess the true risk levels.

In conclusion, ICI retreatment can be safe and effective for select patients with severe irAEs, although careful selection and monitoring by a multidisciplinary team is essential. Larger prospective studies are needed to validate these findings and establish optimal selection and monitoring strategies, ultimately improving outcomes for patients with limited treatment options.

## Supplementary Material

oyaf120_suppl_Supplementary_Material

## Data Availability

The data that support the findings of this study are available from the corresponding author, T.I., upon reasonable request.
