# Supplementary material for: Safety and efficacy of retreatment with immune checkpoint inhibitors after severe immune-related adverse events
Source: Oncologist. 2025 Jun 14;30(6):oyaf120. doi: 10.1093/oncolo/oyaf120 (PMC12166119; doi:10.1093/oncolo/oyaf120)
Supplement: oyaf120_suppl_Supplementary_Material [file oyaf120_suppl_supplementary_material.docx]

**Supplemental Table 　Profiles of the five complete responders to ICI retreatment post-severe irAEs**

| **Age** | **Tumor type** | **Causal ICI** | **ICI retreatment** | **Pre-irAE response** | **Pre/post-irAE ICI interval (days)** | **Time to initial irAE onset (days)** | **Initial irAE** | **Subsequent irAE** | **Retreatment duration (days)** | **Outcome after retreatment** |
| --- | --- | --- | --- | --- | --- | --- | --- | --- | --- | --- |
| 40s | Malignant melanoma | Ipilimumab  +Nivolumab | Surgery  +Pembrolizumab | CR | 110 | 36 | Hepatotoxicity G4 | None | 1484 | Treatment ongoing |
| 40s | Renal cancer | Ipilimumab  +Nivolumab | Nivolumab | SD | 315 | 84 | Hepatotoxicity G3 | None | 420 | Treatment holiday after CR |
| 70s | Renal cancer | Nivolumab | Nivolumab | PR | 77 | 14 | Hepatotoxicity G3,  Dermatitis G3 | None | 511 | Treatment discontinued at patient's request |
| 70s | Head and neck cancer | Nivolumab | Nivolumab | SD | 14 | 112 | Nephritis G3 | Hypophysitis G3 | 867 | Treatment ongoing |
| 60s | Lung cancer | Pembrolizumab | Pembrolizumab | CR | 49 | 415 | Hepatotoxicity G3,  Dermatitis G3 | Dermatitis G3 | 224 | Treatment discontinued due to irAE |

ICI: immune checkpoint inhibitor; irAE: immune-related adverse event; G3, G4: grade 3, grade 4 (severity of adverse events); CR: complete response; PR: partial response; SD: stable disease; PD: progressive
